# Quantitative changes of upper airway in class III patients undergoing bimaxillary surgery after one-year follow-up: a retrospective study

**DOI:** 10.1186/s13005-022-00317-2

**Published:** 2022-04-19

**Authors:** Haizhen Li, Chongke Sun, Yanlong Chen, Zhipeng Sun, Xuemei Gao

**Affiliations:** 1 Department of Orthodontics, Peking University School and Hospital of Stomatology, 22 Zhongguancun South Avenue, Haidian District, Beijing, 100081 People’s Republic of China; 2 Department of Radiology, Peking University School and Hospital of Stomatology, 22 Zhongguancun South Avenue, Haidian District, Beijing, People’s Republic of China

**Keywords:** Upper airway, Orthognathic surgery, CBCT, Class III malocclusions

## Abstract

**Background:**

Bimaxillary surgery is often performed for class III malocclusion, and its complex influence on the upper airway has been well considered. The aim of this research was to provide a scaled formula between upper airway volume changes and bone movements in Class III patients after orthognathic surgery.

**Materials and methods:**

Using a retrospective study design, the investigators enrolled a total of 30 class III malocclusion patients who were undergoing bimaxillary surgery as the study subjects. The subjects included 15 males and 15 females, and their average age was 23.3 ± 3.4 years. CBCT (cone beam tomography) was performed both before and one year after the surgery for each patient. The changes in the soft palate, tongue and upper airway were measured by using CBCT data that was collected before and after surgery. 3D superimposition of CBCT was performed to calculate three-dimensional jaw movements. A multiple regression analysis was used to calculate the quantitative relationship between airway volume changes and jaw movements.

**Results:**

The nasopharynx airway volume was observed to be increased by 1064.0 ± 1336.2 mm^3^, whereas the retropalatal and retroglossal airway volumes were observed to be decreased by 1399.0 ± 2881.6 mm^3^ and 1433.8 ± 3043.4 mm^3^, respectively, after the surgery. One millimetre forward and downward movements of the PNS resulted in increases of 626.90 mm^3^ and 392.18 mm^3^ in nasopharynx airway volume, respectively. Moreover, one millimetre retrogression of the B point caused decreases of 314.6 mm^3^ and 656.6 mm^3^ in the retropalatal and retroglossal airway volume, respectively. The changes in the soft palate contributed to the decrease in the retropalatal airway volume, whereas the tongue compensated for the decrease in the retroglossal airway volume.

**Conclusion:**

The movements of the PNS and B points could be used to predict upper airway volumetric changes in Class III patients after maxillary advancement and mandibular setback.

## Background

Orthognathic surgery is well accepted for patients with skeletal Class III deformities, due to its improvements in facial appearance, mastication function and long-term stability [1]. However, it was recently found that large-scale backwards movement of the mandible may have a negative impact on the airway space [2–5]. The volume reduction of the pharyngeal airway causes increasing concerns because it is considered to be one of the aetiologies of OSA (obstructive sleep apnea) [6]. Additionally, postoperative airway changes are especially considered by oral surgeons when developing the surgery plan.

The complexity of Class III orthognathic surgery involves the fact that maxillary advancement leads to the widening of the upper airway [7, 8], whereas mandible setback results in the narrowing of the upper airway [9–11]. In the real world, the amount of jaw movement and airway changes can vary greatly among different patients. Many previous studies have described airway changes after specific operation methods, but little research has been conducted on the relationship between airway changes and the amount of bone movement [12]. In fact, the quantitative relationship between airway changes and bone movements may be more helpful for surgeons in deciding the surgery design.

In reports about long-term postoperative airway changes, postoperative airway remodelling has been observed to be a gradual process that changes with time [13]. This is supported by evidence that the airway space decreases in the first few months but recovers after a period of time [11]. The reasons for this effect include the postoperative physiological adaptation of soft tissue to the new environment and the location shift of the hyoid bone, as evidenced by some studies [14, 15]. In his study, Saitoh theorized that the connecting soft tissues and the musculature that anchor and stabilize the airway explain how skeletal movements can influence the upper airway [16]. The adaptive changes in soft tissue should be considered an important factor when evaluating airway changes.

Thus, the purpose of this retrospective study focused on the quantitative relationship between hard and soft tissue movements and airway changes in Class III patients at one year after bimaxillary surgery.

## Materials and methods

### Subjects

The protocols were approved by the Medical Ethics Committee of the Peking University School and Hospital of Stomatology (No. 202053015). Due to the retrospective nature of the present study, informed consent was not needed. The sample size of this research was calculated by using PASS (Power Analysis and Sample Size, Utah, USA.) software. The desired value and standard deviation were δ = 724.71 and SD = 1098.38, respectively, which was calculated based on a pre-experiment of ten patients. The other statistical settings were α = 0.05 and β = 0.1. At least 27 samples were needed for this study.

The patients were recruited from the Department of Radiology, Peking University School of Stomatology from January 2017 to December 2019. The inclusion criteria included skeletal Class III deformities (ANB < 0°; overjet<− 2 mm), performance of maxillary advancement and mandibular setback and genioplasty procedures and sufficient visual fields of images. The exclusion criteria included congenital syndromes, diagnoses of temporomandibular joint disorders, BMI > 35 kg/m^2^, records with respiratory disease or a history of facial trauma. Patients were also excluded if the change in cranio-cervical inclination was greater than 5° between the T1 (one week before surgery) and T2 (an average of 1 year after surgery) scans, which was done to avoid significant influences of neck extension on airway measurements [7, 17].

In total, thirty patients, including 15 males and 15 females, were selected. All of the patients were aged from 18 to 29 years, and the average age was 23.3 ± 3.4 years. Moreover, the average BMI of the patients was 21.49 ± 3.92 kg/m^2^.

### Cone beam computed tomography (CBCT) scanning

CBCT scans were obtained at 2 time points: T1 (one week before surgery) and T2 (an average of 1 year after surgery). All of the subjects were scanned with the same machine (DCT PRO; Vatech Co, Seoul, South Korea) in routine processes. For each patient, CBCT was performed according to the international guidelines. The CBCT settings were as follows: tube voltage, 90 kVp; scan time, 24 s; tube current, 4.0 mA and voxel size, 0.3 mm. The data were imported into Dolphin software version 11.0 (Dolphin Imaging and Management Solutions, Chatsworth, California USA) for data processing [18].

### Three-dimensional coordinate system and image superimposition

**Fig. 1:**
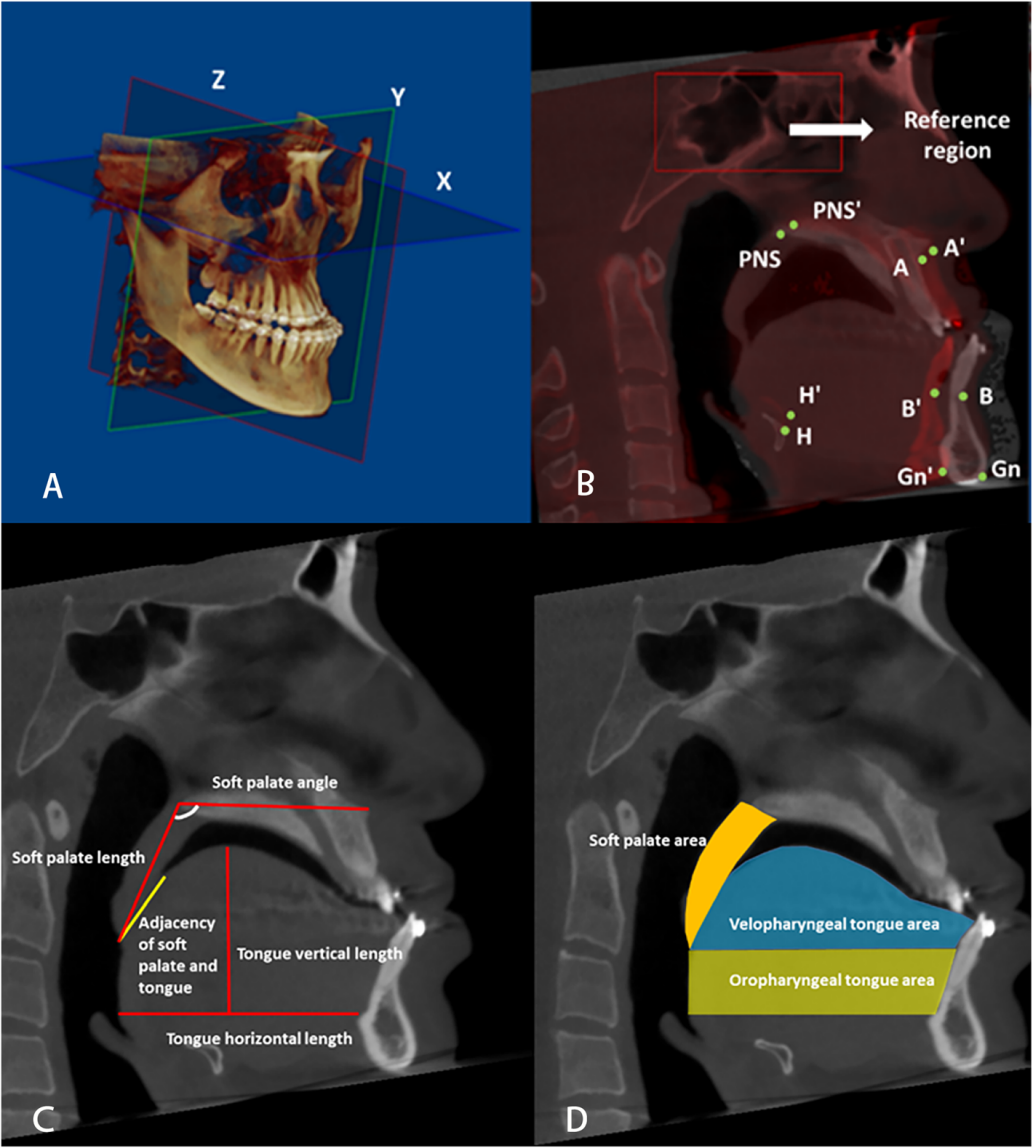
Three-dimensional coordinate system and image superimposition of the CBCT. **A**. Three-dimensional coordinate system. **B**. Superimposition of preoperative and postoperative CBCT. The red box stood for the reference region. The definition landmarks were given in Table 2. **C**. Linear measurements of soft palate and tongue. **D** Area measurements of soft palate and tongue

A three-dimensional coordinate system was set up for each CBCT image (Fig. 1A). The Frankfort horizontal plane was set as the x-coordinate, and the left side direction was recorded as being positive. A plane perpendicular to the x-axis and passing through Nasion was defined as the y-coordinate, and the upwards direction was recorded as being positive. The plane perpendicular to the other planes that went through Nasion was defined as the Z plane, and the forward direction was recorded as being positive.

Quantifications of jaw movements were performed via the superimposition of preoperative and postoperative CBCT images. Via the Dolphin software, voxel-based superimposition was performed as previously described by Bazina, Mohamed and Hernando [19, 20]^.^ The anterior cranial base was selected as the reference region, which is shown in Fig. 1B.

Five landmarks, including PNS, A, B, Gn and H, were digitized on the CBCT images to represent the posterior maxilla, anterior maxilla, anterior mandible, chin and hyoid bone, respectively. We digitized the landmarks in the sagittal, coronal and axial planes according to the method previously described by Ludlow JB [21]. The definitions of the landmarks are provided in Table 1, and a diagrammatic sketch is shown in Fig. 1B. The coordinates of the landmarks were exported by the software. The movements of the jaws were calculated by subtracting the coordinates both before and after the surgery.

**Table 1:** Definition of the landmarks, planes and airway measurements used in the research

	Definition
**A point**	Deepest anterior point in concavity of anterior maxilla
**B point**	Deepest anterior point in concavity of anterior mandible
**Gn point**	The most anterior and inferior point of the chin
**N point**	Most anterior point of frontonasal suture in midsagittal plane
**Airway height**	The distance from roof of the airway to the upper border of larynx
**Total airway volume**	Airway extending from roof of the airway to the upper border of larynx
**CSA**	Cross-sectional area of pharyngeal airway section
**APL**	Antero-posterior length of pharyngeal airway section
**LTW**	Largest transverse width of pharyngeal airway section
**Hard palate plane**	Plane parallel to FH plane and passing through most posterior point of hard palate
**Soft palate plane**	Plane parallel to FH plane and passing through the middle point of soft palate and PNS
**Tongue plane**	Plane parallel to FH plane and passing through the middle point of soft palate and epiglottis point.
**Epiglottis plane**	Plane parallel to FH plane and passing through Most posterior point of epiglottis
**Soft palate length**	The distance from PNS to the top of soft palate
**Soft palate angle**	The angel formed by soft palate and hard palate
**Horizontal tongue length**	The horizontal distance from the last point of the tongue to the internal border of the mandible
**Vertical tongue length**	The distance from the top of the tongue to the epiglottis plane

### Soft tissue and airway measurements

All soft tissue measurements were measured in the middle sagittal plane under the same threshold settings, including the length, area and angle of the soft palate, as well as the horizontal length and vertical height of the tongue (Fig. 1C). To describe the relationship between the soft palate and tongue, their contact distance was recorded. The tongue was divided into the velopharyngeal tongue and oropharyngeal tongue, according to the plane that passed through the tip point of the soft palate, and the area of the two parts was calculated (Fig. 1D).

**Fig. 2:**
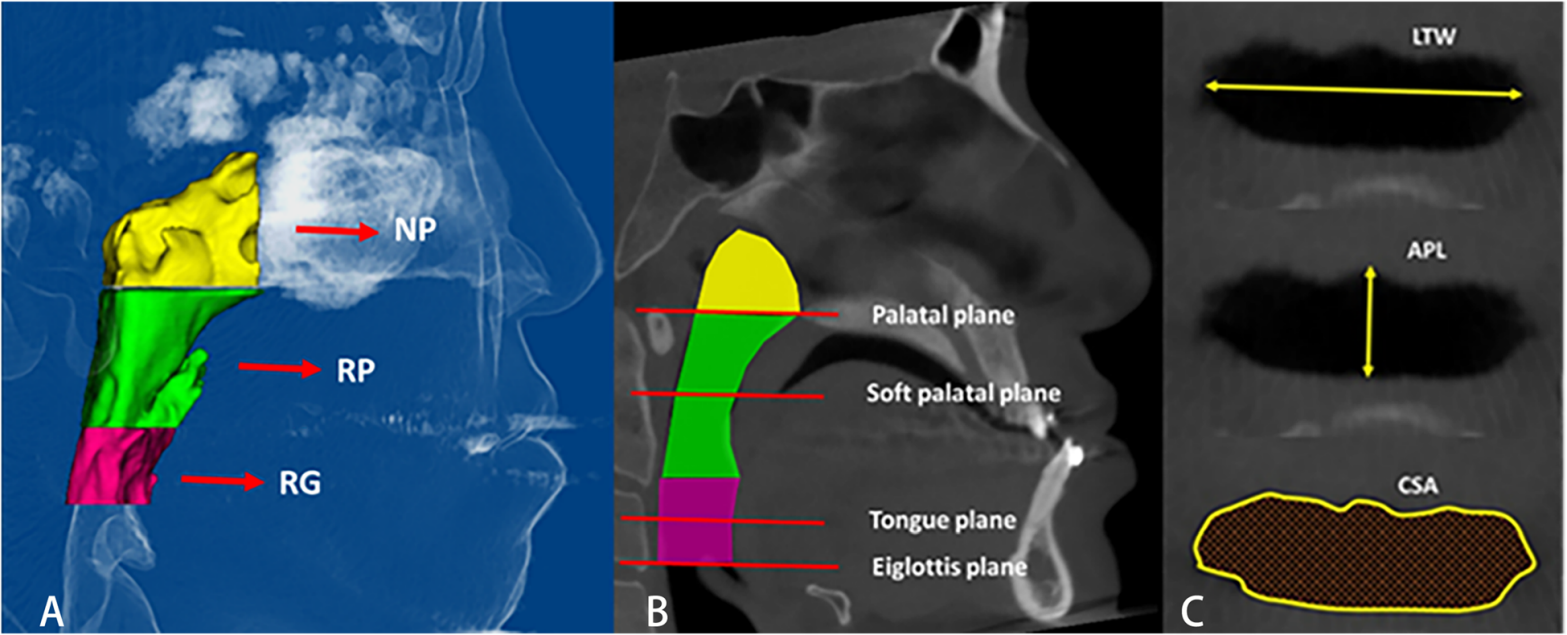
The three-dimensional diagrammatic sketch of upper airway division and their illustrations. **A**. Division of the pharyngeal airway in the midsagittal plane. NP: Nasopharynx airway, RP: Retropalatal airway, RG: Retroglossal airway. **B**. Four planes at the levels of the hard palatal, soft palate, tongue, and epiglottis. **C**. Anteroposterior length and transversal width, cross-sectional area at each plane

The total airway was defined as the sum of the nasopharynx, retropalatal and retroglossal airways, which are illustrated by diagrammatic sketches in Fig. 2 A and B. The nasopharynx airway was defined as the area between a plane parallel to the Frankfort horizontal through the PNS and a plane perpendicular to the Frankfort horizontal through the PNS [22]. The retropalatal airway was defined as the airway that was superiorly bounded by the plane parallel to the Frankfort horizontal passing through PNS and inferiorly bounded by a plane parallel to the Frankfort horizontal passing through the top of the soft palate [22]. The retroglossal airway was defined as the airway that was superiorly bounded by the lower border of the retropalatal airway and inferiorly bounded by a plane parallel to the Frankfort horizontal passing through the top of the epiglottis.

The airway was 3-dimensionally scanned to ensure that every aspect was included. The volume was calculated via Dolphin software under the same threshold settings. To evaluate the airway changes at different levels, we measured the anteroposterior length (APL), largest transverse width (LTW) and cross-sectional area (CSA) at 4 axial planes (palatal plane, soft palate, tongue and epiglottis) (Fig. 2C) [23]. The definitions of the airway measurements are provided in Table 1.

The operator reliabilities of landmarks and airway measurements were evaluated by using intraclass correlation coefficients. Ten subjects were randomly selected from all of the subjects to test for intraoperator and interoperator reliabilities. Intraoperator reliability was performed by the same operator (HZ. L.), who defined the landmarks twice in one week. Interoperator reliability was assessed by 2 operators (HZ. L and YL. C) at the same time. The intraoperator reliability coefficients varied between 0.77 and 0.99. The interoperator reliability coefficients ranged from 0.79 to 0.99.

### Statistical analyses

SPSS software (Windows version 17.0; SPSS Inc. Chicago, IL, USA) was used to perform the data analysis. The data were described as the mean and standard deviation after using the Shapiro–Wilk test to identify a normal distribution. Paired t tests were used to verify significant differences in the airway measurements and in hard and soft tissue changes. A single-factor linear regression analysis was conducted to screen the variables (included if *p* < 0.1). Subsequently, stepwise multiple linear regression analyses were performed to identify the factors that affected the airway volume.

## Results

### Maxillary and mandibular movements

Three-dimensional movements of landmarks are provided in Table 2. The main changes occurred in the sagittal dimension, with the A point and PNS point moving forward and the B point and Gn point moved backwards.

**Table 2:** The mean and standard deviation of landmark movements used in the research (mm)

Mean + SD	PNS	A	B	Gn
Horizontal movements	−0.2 ± 0.9	0.1 ± 0.9	−0.2 ± 3.2	− 0.2 ± 4.4
Vertical movements	1.3 ± 1.6	−0.4 ± 1.6	1.8 ± 3.1	2.6 ± 3.8
Sagittal movements	4.1 ± 1.2	4.1 ± 1.4	−5.7 ± 2.8	−4.6 ± 3.6

### Soft tissue and hyoid bone changes

The soft tissue and hyoid changes after orthognathic surgery was shown in Table 3. The length of the soft palate was observed to be larger than that before the operation. The included angle between the soft palate and hard palate was also found to be larger than that before the operation. The tongue arched up from the horizontal into the vertical planes due to mandible compression, with the horizontal length decreasing and vertical length increasing. The contact distance of the soft palate and tongue were also observed to be significantly increased. Moreover, the three-dimensional positions of the hyoid bone did not significantly change.

**Table 3:** The soft tissue and hyoid changes after orthognathic surgery in thirty skeletal Class III patients

	Pre-surgery	Post-surgery	Difference	P
**Soft palate length(mm)**	33.2 ± 3.2	35.2 ± 4.2	2 ± 3.3	0.003**
**Soft palate angle**	109.2 ± 7.7	116.2 ± 6.8	7 ± 6.1	0.000***
**Soft palate area(mm** ^**2**^ **)**	246.7 ± 59.7	260.5 ± 57.1	13.8 ± 46.9	0.118
**Tongue horizontal length(mm)**	58.1 ± 5.4	53.6 ± 5.3	−4.5 ± 3.5	0.000***
**Tongue height(mm)**	39.5 ± 6.2	43.6 ± 4.8	4.5 ± 3.4	0.000***
**Retropalatal tongue area(mm** ^**2**^ **)**	845.5 ± 321.1	973.1 ± 271.6	127.6 ± 274.1	0.016*
**Retroglossal tongue area(mm** ^**2**^ **)**	1117.5 ± 306	1193.8 ± 264.5	76.3 ± 222	0.070
**Horizontal hyoid bone coordinates(mm)**	0.4 ± 3.2	−0.4 ± 2.3	−0.8 ± 2.5	0.095
**Vertical hyoid bone coordinates(mm)**	−83.8 ± 8.6	−82.8 ± 10.2	1.0 ± 4.7	0.259
**Sagittal hyoid bone coordinates(mm)**	6.2 ± 9.9	5.8 ± 9.1	−0.4 ± 2.9	0.491

Paired *t* test: *p < 0.05, **p < 0.01, ***p < 0.001

### Airway measurements

An increase in the nasopharynx airway space and a decrease in the retropalatal and retroglossal airway spaces were observed (Table 4). A significant decrease was also observed in the cross-sectional area at the levels of the soft palate, tongue, and epiglottis. The exception was the cross section at the level of the hard palate plane, with an increase in the APL (*p* < 0.05); however, the area and LTW of this section did not significantly change.

**Table 4:** The airway changes after orthognathic surgery in thirty skeletal Class III patients

	pre-surgery mean ± SD	A year post-surgery mean ± SD	Differences mean ± SD	***P***
**Volume(mm**^**3**^**)**				
Total airway volume	29,651.9 ± 10,143.7	28,138 ± 9004.6	− 1513.9 ± 4341.4	0.066
Nasopharynx airway	9333.2 ± 2883.8	10,346.7 ± 3566.8	1013.5 ± 1348.2	0.000***
Retropalatal airway	13,496.9 ± 5432.6	12,121 ± 4574.5	− 1375.9 ± 2403.4	0.004**
Retroglossal airway	6821.7 ± 3925.2	5670.3 ± 2729.7	− 1151.4 ± 2660.7	0.025*
**Cross section area(mm2) at the level of**				
Palatal	650 ± 170	653.5 ± 184.8	3.5 ± 95.2	0.841
Soft palatal	422.8 ± 150.6	390.3 ± 137.3	−32.5 ± 77.4	0.029*
Tongue	375.5 ± 145.6	303.3 ± 122.3	−72.1 ± 98	0.000***
Epiglottis	359.9 ± 129.8	301.5 ± 99.6	−58.3 ± 101	0.004**
**Width(mm) at the level of**				
Palatal plane	33 ± 5	32.8 ± 5.3	−0.3 ± 2.6	0.582
Soft palatal	34.8 ± 7.7	31.6 ± 6.2	−3.2 ± 4.9	0.001**
Tongue	32.1 ± 4.3	29.2 ± 4.8	−2.9 ± 1.7	0.000***
Epiglottis	32.5 ± 4.2	31.3 ± 3.8	−1.2 ± 2.2	0.007
**Anteroposterior length (mm) at the level of**				
Palatal plane	21.1 ± 4.2	23.2 ± 4.4	2.1 ± 2.5	0.000***
Soft palatal	15.7 ± 2.8	14.5 ± 3	−1.2 ± 1.1	0.000***
Tongue	14.3 ± 3.6	12.8 ± 3.7	−1.5 ± 3.1	0.012 *
Epiglottis	14.2 ± 3.9	12.7 ± 3.3	−1.5 ± 3.3	0.019 *

Paired *t* test: *p < 0.05, **p < 0.01, ***p < 0.001

### Multiple linear regression analysis

**Fig. 3:**
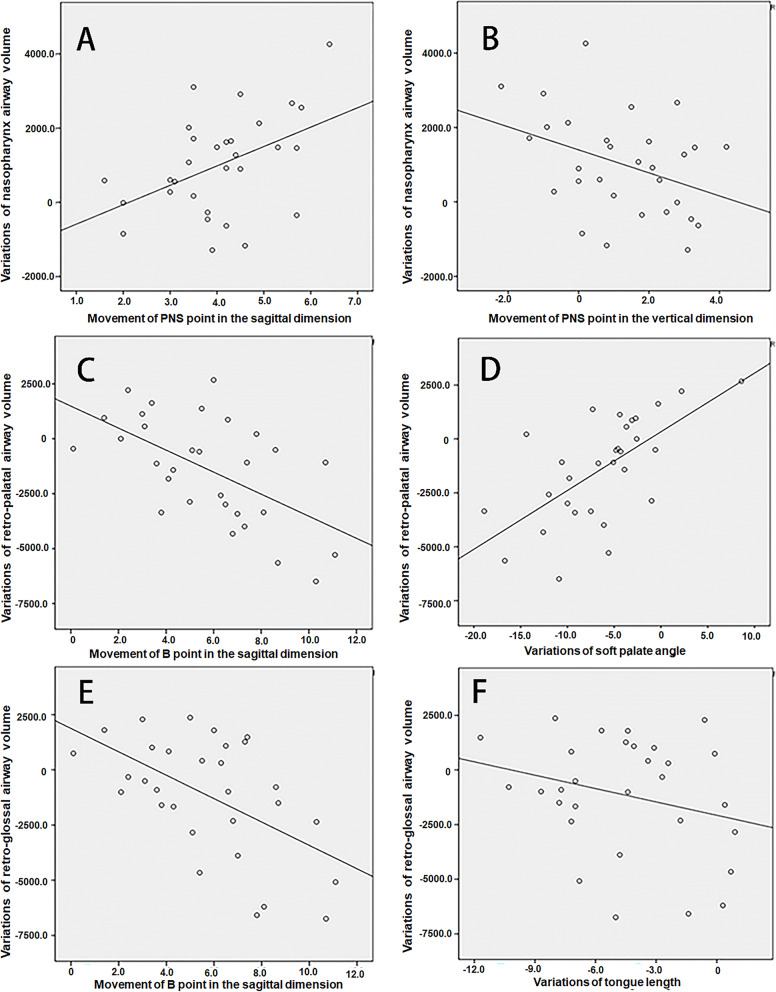
Single linear analysis of the variables bone movements and volume variation. **A**. PNS horizontal movement and nasopharynx airway volume (r = 0.451). **B**. PNS vertical movement and nasopharynx airway volume (r = 0.378). **C**. B horizontal movement and retropalatal airway volume (r = 0.574). **D**. Soft palate angle and retropalatal airway volume (r = 0.643). **E**. B horizontal movement and retroglossal airway volume (r = 0.548). **F**. Tongue horizontal length and retroglossal airway volume (r = 0.261)

The scatter plots of the included factors are shown in Fig. 3. The results of the multivariate linear regression analysis are provided in Table 5. The nasopharynx airway volume was shown to be affected by PNS movement; one millimetre of forward movement of the PNS point increased the airway volume by 626.90 mm^3^, whereas one millimetre of upwards movement decreased the airway volume by 392.18 mm^3^. Furthermore, retro-palatal airway volume was observed to be affected by B sagittal backwards movement and the soft palate, with 314.6 mm^3^ and 204.9 mm^3^ volume reductions observed per unit, respectively. The retroglossal airway was shown to be affected by B sagittal movement and tongue horizontal length; one millimetre setback of the former factor reduced airway volume by 656.6 mm^3^, whereas one millimetre reduction of the latter factor offset airway volume by 356.4 mm^3^. To better explain the effects of jaw movement on the airway, the quantitative relationship between jaw movement and the percentage change of airway was also calculated, and the results are provided in Table 5.

**Table 5:** Multiple linear regression analysis between the surrounding structures and airway changes

Y	Variables(Δ = Pre-Post)	Linear regression coefficient	P	Multivariate ANOVA	R^2^
Nasopharynx airway volume	PNS horizontal movement	626.9	0.001	0.001 **	0.424
	PNS vertical movement	−392.2	0.003		
	Constant	− 1040.2	0.158		
Nasopharynx airway volume percentage	PNS horizontal movement	0.059	0.005	0.002 **	0.367
	PNS vertical movement	−0.042	0.005		
	Constant	−0.077	0.342		
Retropalatal airway volume	Soft palate angle	204.9	0.003	0.000***	0.518
	B sagittal movements	−314.6	0.022		
	constant	1710.7	0.029		
Retropalatal airway volume percentage	Soft palate angle	0.014	0.006	0.000***	0.477
	B sagittal movements	−0.021	0.034		
	constant	0.118	0.036		
Retroglossal airway volume	B sagittal movements	−656.6	0.000	0.000***	0.491
	Tongue horizontal length	356.4	0.004		
	Constant	992.6	0.271		
Retroglossal airway volume percentage	B sagittal movements	−0.046	0.005	0.007**	0.306
	Tongue horizontal length	−0.072	0.022		
	Constant	0.143	0.349		

* *p* < 0.05, **p < 0.01, ***p < 0.001

## Discussion

This study aimed to provide a mathematical calculation to predict the volumetric changes in the upper airway after orthoganthic surgery in Class III patients, which will benefit the estimation and prediction of potential sleep-disordered breathing risks in the preoperative planning stage. The risk has been previously described by a systematic review that showed that reduction of the upper airway should be considered for patients who have undergone large mandible setback surgery [24]. However, two-jaw surgery is more common than single-jaw surgery for Class III patients in the real world for the sake of a better profile. Some scholars believe that two-jaw surgery is beneficial to the airway [25–28], whereas other scholars hold the opposite view [3, 29–31]. The divergence among the studies not only resulted from different surgical designs but was also due to the degrees of bone movement of each surgery [32]. Therefore, a formula established by using regular movement of landmarks of the maxilla and mandible could be significant for evaluating airway changes.

Wiedemeyer used a mathematical method to predict airway changes in Class II patients [33], which was a valuable attempt at practicing precision medicine. However, his research focused on single jaw surgery, and the lateral cephalometric radiograph that was used in his study could not reflect the actual 3D airway structures. Three-dimensional digital technology based on CBCT was used in this study to observe airway, jaw and soft tissue changes from a three-dimensional perspective. Additionally, one year was set as the observation window in consideration of time, in order to avoid the recurrence stage and to obtain more stable results. According to previous studies, more than 6 months was considered the stable stage for Class III organathic surgery patients [11, 30].

From the current study, it could be observed that one millimetre forward and downwards movements of PNS resulted in an increase of 626.90 mm^3^ and 392.^18^ mm^3^ of the nasopharynx airway volume, respectively. Subsequently, for one millimetre retrogression of point B, a 314.6 mm^3^ decrease in the retropalatal airway and a 656.^6^ mm^3^ decrease in the retroglossal airway could be seen. There have been some similar reports that have proven the relationship between upper airway changes and the amount of bone movement. For example, Mohammed found that nasopharynx airway changes were related to the A point [7]. Additionally, Hart demonstrated the quantitative relationship that one millimetre of forward or backwards movements of the D point could account for a 403.6 mm^3^ increase in the total airway or a 383.9 mm^3^ decrease in the oropharyngeal airway volume [34]. However, Brunetto suggested the use of percentage values instead of absolute values [35]. The experimental results may vary among studies because of different surgical designs, sample characteristics, measurement indices and follow-up times.

According to the present study, the main disadvantage factor for upper airway changes was mandible setback, which not only caused an encroachment of the pharynx but also pushed the soft palate back to the retropalatal airway space, which ultimately decreased the airway volume. Moreover, the advantageous factors for upper airway changes included maxilla movement and tongue adaptation changes. The forward and downwards movements of the maxilla acted as a favourable factor for the postoperative airway changes due to its positive correlation with the nasopharynx airway volume. In addition to this, adaptive tongue changes (arched dorsum) were also observed to be helpful for the upper airway. A multivariate linear regression analysis showed that the decrease in tongue horizontal length could offset retroglossal airway volume reduction. The tongue played a compensatory role in mandible retraction if given a relatively long time to adapt to the new jaw position.

In addition, the changes in the hyoid bone did not significantly change in this study, which was consistent with the studies by Aydemir [36].

As a retrospective study, the present study had limitations due to several missing data details. The reference data provided in the present study may only be used in Asian Class III patients undergoing bimaxillary surgery. Furthermore, the conclusion of this study was drawn from young and thin patients, which may not act as guidelines for patients with larger BMIs, older ages or populations from other races. In addition, a one-year period was set as the observation window, which could not reflect even longer changes in the airway. Finally, this study was based on sitting CBCT images and a lack of a polysomnography (PSG) index, which could provide neither sleep functional data nor direct evidence of OSA risks.

## Conclusion

This was a pilot study to provide a quantitative relationship between airway volume changes and bone movements. The upper airway volume was comprehensively affected by both maxilla advancement and mandible recession. The compensatory performance of the tongue also played an advantageous role in one-year airway changes.

## Data Availability

All data generated or analyzed during this study are included in this published article.

## Abbreviations

CBCT: Come beam tomography
OSA: Obstructive sleep apnea
